# The Effect of Vitamin D3 Injection Combined With High-Intensity Interval Training on Excessive Autophagy in the Heart Tissue of Type 2 Diabetes–Induced Rats: An Analysis of the mTOR–Beclin-1–Fyco-1–Cathepsin D Pathway

**DOI:** 10.1155/cdr/8817195

**Published:** 2025-03-19

**Authors:** Hadi Golpasandi, Mohammad Rahman Rahimi

**Affiliations:** Department of Exercise Physiology, Faculty of Humanities and Social Sciences, University of Kurdistan, Sanandaj, Iran

**Keywords:** autolysosomal fusion, excessive autophagy, HIIT, Type 2 diabetes, vitamin D3

## Abstract

**Introduction:** This study investigated the effect of vitamin D3 injection combined with high-intensity interval training on cell signaling pathways involved in excessive autophagy, specifically the mTOR (mechanistic target of rapamycin)–Beclin-1–Fyco-1 (FYVE and coiled-coil domain-containing protein 1)–cathepsin D pathway, in the heart tissue of Type 2 diabetes–induced rats.

**Method:** In this experimental study, 32 male Wistar rats were fed a high-fat diet for 6 weeks to induce Type 2 diabetes, followed by a single subcutaneous injection of 35 mg/kg streptozotocin (STZ). The rats were then randomly assigned to one of four groups: (1) diabetes control (DC), (2) diabetes + HIIT (DT), (3) diabetes + vitamin D3 (DV), and (4) diabetes + HIIT + vitamin D3 (DTV). HIIT sessions were conducted for 8 weeks, five times per week, at an intensity of 85%–95% of maximum running speed (*V*_max_), while vitamin D3 was administered weekly via subcutaneous injection at a dose of 10,000 IU/kg. Twenty-four hours after the intervention period, heart and left ventricular tissues were collected for analysis of the levels of autophagy signaling proteins mTOR, phosphorylated mechanistic target of rapamycin (pmTOR), Beclin-1, Fyco-1, and cathepsin D.

**Results:** Two-way ANOVA revealed that Type 2 diabetes significantly increased the levels of Beclin-1, Fyco-1, and cathepsin D (*p* < 0.001) while significantly reducing the levels of mTOR and pmTOR (*p* < 0.001). HIIT, vitamin D3 injection, and their combined treatment significantly decreased the levels of Beclin-1, Fyco-1, and cathepsin D and increased the levels of mTOR and pmTOR compared to the diabetes control group (*p* < 0.001).

**Conclusion:** Type 2 diabetes increases autophagy in the left ventricle, marked by altered levels of key autophagy proteins. HIIT and vitamin D3 injections mitigate these effects by enhancing mTOR signaling and reducing excessive autophagy. These interventions show promise as nonpharmacological strategies to improve cardiac health in Type 2 diabetes and could be incorporated into clinical and rehabilitation programs.

## 1. Introduction

Type 2 diabetes (T2DM) is a chronic metabolic disorder characterized by insulin resistance (IR) and impaired insulin secretion, leading to hyperglycemia [1]. Emerging research has highlighted the role of autophagy and lysosomal function in the development and progression of T2DM [2]. Autophagy, a cellular degradation process, is crucial for maintaining cellular homeostasis by removing damaged organelles and proteins. In T2DM, impaired autophagy in pancreatic *β* cells leads to decreased insulin secretion and beta cell apoptosis [3], which can be improved by increasing autophagy [4]. In T2DM, lysosomal function is often impaired, which leads to the accumulation of autophagic substrates, and this can exacerbate metabolic dysfunction by increasing oxidative stress and inflammation [5]. However, lysosomal disorders, which disrupt the degradation capacity of lysosomes, also play a role in the pathology of T2DM [6]. Lysosomes are essential for the final breakdown of autophagic cargo, as their enzymes degrade macromolecules into building blocks that are then recycled for cellular processes. The mechanistic target of rapamycin (mTOR) acts as a negative regulator of autophagy and lysosomal biogenesis. In T2DM, mTOR overactivation inhibits autophagy, while lysosomal dysfunction can further regulate mTOR signaling through feedback mechanisms [7]. A previous study showed that T2DM led to a decrease in mTOR protein levels in the heart tissue of rats with induced T2DM. This decrease was accompanied by an increase in markers involved in phagosome–lysosome fusion, including FYVE and coiled-coil domain-containing protein 1 (Fyco-1) and cathepsin D (CTSD) [8]. However, the relationship between mTOR and lysosomal markers has yet to be thoroughly investigated. Fyco-1 plays a key role in the transfer and maturation of autophagosomes to lysosomes. Its activity is regulated by nutrient status and cellular energy levels and is closely linked to metabolic processes [9, 10]. CTSD is a lysosomal aspartic protease involved in protein degradation and is essential for maintaining protein homeostasis and processing autophagic cargo [11, 12]. Studies have shown that CTSD expression is elevated in the adipose tissue, liver, and heart of individuals with T2DM, which is associated with IR and impaired glucose metabolism [13–15].

Exercise training has always been known as one of the nondrug strategies to improve insulin sensitivity, glucose metabolism, and overall metabolic health in people with T2DM [16]. Exercise training can regulate the expression and activity of CTSD and thus improve autophagic flux and cell homeostasis, which can improve metabolic control and reduce inflammation in diabetics [17]. In this regard, it has been reported that exercise training with different intensities causes the regulation of autophagy [18, 19]. However, there are limited studies on the effects of various exercises on the levels of proteins related to lysosomal markers. High-intensity interval training (HIIT) has been shown to affect lysosomal markers, which are critical for cellular waste disposal and recycling processes, including autophagy. It has recently been reported that 8 weeks of HIIT stimulated autophagy through activation of the ERK/P90SRK pathway and inhibition of the mTOR/P70S6K pathway, leading to improvement of IR and reduction of blood glucose [20]. Although research has mainly identified resistance training as a potent stimulator of mTOR in skeletal muscle [21], it has recently been reported that HIIT may regulate metabolic and mechanical pathways through mTOR by placing high stress on the heart [22, 23], so further research in this area could provide new insights into the tissue-dependent role of mTOR.

Lysosomes are involved in breaking down damaged or unnecessary cellular components and their activity is essential to maintain cellular homeostasis, especially in response to stress caused by vigorous physical activity [24]. HIIT enhances the expression of key autophagy markers such as LC3-II, ATG-3, and Beclin-1 in skeletal muscle, indicating increased autophagic flux. These changes contribute to improved mitochondrial function and metabolic adaptations, which are essential for maintaining cellular homeostasis and preventing metabolic disorders [24]. On the other hand, another study showed that moderate-intensity continuous training (MICT), with or without vitamin D3 supplementation, decreased autophagy markers [8]. It has been reported that vitamin D + vitamin D receptor (VDR) interaction can restore impaired autophagy in kidney tissue of streptozotocin (STZ)-induced diabetic rats, which can be attributed to the activation of the Ca2+–CAMKK2–AMPK pathway in renal tubular epithelial cells [25]. Vitamin D3, also known as cholecalciferol, plays an important role in regulating autophagy and lysosomal markers. Several studies have demonstrated the multifaceted effects of vitamin D3 on cellular processes, including its effect on autophagy, a critical mechanism for maintaining cellular homeostasis and responding to metabolic stress [26, 27]. In a study, the interaction of HIIT with vitamin D3 injections was shown to reduce levels of ULK-1 and subsequently reduce inflammation in rats with T2DM [28], although some of them have reported the effects of increasing autophagy [29, 30] and some decreasing autophagy [8].

In this study, we used the animal model, male Wistar rats, due to their physiological similarity to humans, as this animal model has been widely used in metabolic research. In the present study, we used the combination of high-fat diet (HFD) + STZ injection, which is a model for the development of metabolic disorders, to induce T2DM, which in this case provides insights into the potential therapeutic effects of HIIT and vitamin D3 on diabetic cardiomyopathy by investigating the molecular mechanisms of autophagy and lysosomal regulation in the heart of rats induced with T2DM.

The initial hypothesis of the present study was that HIIT and vitamin D3 supplementation would separately reduce excessive autophagy in the left ventricle induced by T2DM. Furthermore, we hypothesized that the combination of HIIT and vitamin D3 would have an additive effect, leading to a more pronounced increase in mTOR signaling and a reduction in autophagy markers compared with either intervention alone. This hypothesis is based on evidence suggesting complementary mechanisms through which exercise training and vitamin D regulate autophagy pathways. Therefore, this study investigated the interactive effects of HIIT and vitamin D3 injection on cell signaling pathways involved in excessive autophagy, specifically the mTOR–Beclin-1–Fyco-1–CTSD pathway, in the heart tissue of T2DM-induced rats.

## 2. Methods

The present study was experimental and approved by the Research Ethics Committee of University of Kurdistan (Ethics Code IR.UOK.1400.015). All procedures in this study followed the ARRIVE guidelines. Forty-five male Wistar rats, aged 6–8 weeks and weighing a mean of 220 g, were obtained from the Pasteur Institute in Karaj and transferred to the animal laboratory at the Faculty of Basic Sciences, Kurdistan University. The rats were housed in groups of four in transparent polycarbonate cages within a room maintained at 22°C ± 2°C on a 12-h light/dark cycle, with free access to drinking water. Following 1 week of adaptation to the new environment on a standard diet, the rats were randomly divided into two main groups: a normal diet group (*n* = 8) and a HFD group (*n* = 37).

### 2.1. Induction Model of T2DM in Rats Using the Combined Method of HFD and STZ

T2DM was induced using a combination of a HFD and STZ. Rats in the HFD group were fed a HFD for 4 weeks [31], with approximately 182 g of food provided per cage every 24 h. After this period, following a weight measurement, a single dose of STZ (Sigma, Batch Number 0130S, Germany) was prepared at 35 mg/kg in a 0.1 mol/L citrate buffer solution (pH 4.5) and injected intraperitoneally after a 6-h fast [32]. For the standard diet group, a solution of sodium citrate and citric acid was injected as a control [33].

**Figure 1:**
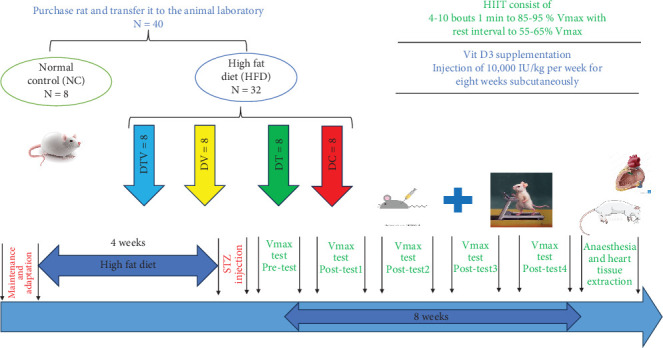
The general schematic of the implementation stages of the study.

Seventy-two hours after STZ injection, the rats' fasting blood glucose levels (after an 8-h fast) were measured using an Accu-Chek Performa glucometer (Germany) to confirm diabetic status [34]. Rats with a blood glucose level over 250 mg/dL (equivalent to above 13.9 mmol/L) were classified as Type 2 diabetic [35], meeting the inclusion criteria for the study; five rats with lower blood glucose levels were excluded. Normal blood glucose was defined as 80–100 mg/dL, which was the range for the normal control group (Figure 1).

### 2.2. Animal Grouping

Rats with induced T2DM continued on the HFD for the remainder of the study and were randomly assigned to one of four groups: (1) diabetic control (DC, *n* = 8); (2) diabetes + HIIT (DT, *n* = 8); (3) diabetes + vitamin D3 (DV, *n* = 8); and (4) diabetes + HIIT + vitamin D3 (DTV, *n* = 8) (Figure 1). Randomization of the rats was performed to ensure unbiased group allocation. Each rat was tagged on its back for identification and assigned to groups using an online randomization tool (https://randomitzer.org/). The sample size of this study was determined using G⁣^∗^Power software (version 3.1.9). Two-way analysis of variance (ANOVA) was used to compare means between groups. Parameters included in the calculation included an expected effect size of 0.40, a significance level of 0.05, a statistical power (1 − *β*) of 0.80, and five experimental groups. Based on these values, a total sample size of 80 animals (16 animals per group) was obtained. However, this sample size was adjusted to comply with the principles of replacement, reduction, and refinement (3R), considering ethical considerations and practical limitations in animal research [36].

### 2.3. Familiarization Phase With Training, *V*_max_ Test, and HIIT Protocol

After dividing the rats into different groups, the rats assigned to the training group DT and DTV were subjected to a familiarization phase in which the rodents walked on a treadmill at a speed of 10–12 m/min for 15 min. During the familiarization week, the treadmill was set to a 0° incline. After that, a graded exercise test (GXT) was performed to determine maximal running speed and time to exhaustion (TTE). The test consisted of 10 stages, each stage lasting 3 min, and the speed increased by 5 m/min in each stage, starting from 5 m/min, and the gradient was considered zero in all stages [37]. The speed at which rats could no longer continue was recorded as their maximum running speed (*V*_max__)_, and TTE was calculated based on the time taken to reach exhaustion. The GXT was repeated in five phases: pretest, Post 1, 2, 3, 4, and 5.

The HIIT protocol was performed during 8 weeks, 5 sessions/week, and each session was 12–30 min with an intensity of 85%–95% *V*_max_ (Figure 1). To maximize adaptation based on the principle of overload, the HIIT program progressed over 8 weeks. During the first and second weeks, rats performed four bouts of 2-min intervals at 85% *V*_max_. The number of intervals gradually increased each week, reaching 10 bouts of 2-min intervals at 95% *V*_max_ by the seventh week. In the eighth week, both intensity (95% *V*_max_) and duration (2-min intervals) were maintained constant to allow adaptation stabilization [38] (Table 1).

### 2.4. Vitamin D3 Injection

For 8 weeks, rats in the vitamin D3 group received weekly subcutaneous injections of vitamin D3 (prepared by Caspian Pharmaceutical Company, Rasht, Iran) (Figure 1). The supplement was prepared by extracting 10,000 IU/kg from a 300,000 IU/kg ampoule using a 1-mL insulin syringe and then diluting it with 5 cc of sesame oil and storing it in tubes at 20°C at room temperature. The rats' weight was measured using a digital scale to determine the appropriate injection dose. A dose of 10,000 IU/kg of the diluted vitamin D3 solution was administered weekly to the DV and DTV groups [39]. The vitamin D3 injections were injected once per week in the morning between 8:00 AM and 10:00 AM to ensure consistency and alignment with diurnal variations in metabolic activity. The injections were given on a nontraining day to prevent any potential interference with the acute effects of exercise on metabolic and physiological responses.

### 2.5. Anesthesia Process, Sampling, and Tissue Removal in Rats

Twenty-four hours after the final aerobic exercise and vitamin D3 interventions and following an 8-h fast, the rats were anesthetized with a subcutaneous injection of ketamine (75 mg/kg, Stroop, Belgium, Batch Number 200178) and xylazine (10 mg/kg, Nixgen, United States, Batch Number 200178). After administering the anesthesia, each rat was left undisturbed for 5 min in a calm environment to minimize stress. Once the rats were confirmed to be fully anesthetized, based on assessments of anesthesia depth (e.g., absence of reflexes and lack of response to stimuli), they were placed on a dissecting board. After confirming deep anesthesia (absence of reflexes and no response to stimuli), euthanasia was performed by decapitation using a guillotine [40]. However, this method was chosen with minimal suffering. The chest was then opened, and blood was drawn directly from the left ventricle of the heart.

### 2.6. Heart Tissue Sampling

When reflexes were absent, euthanasia was performed in accordance with ethical guidelines. The thoracic cavity was then opened through a midline incision, and the heart was rapidly removed and rinsed in cold phosphate-buffered saline (PBS) to remove excess blood and prevent tissue destruction. The left ventricle was carefully dissected and separated from other cardiac tissues to ensure specificity of the mTOR–Beclin-1–Fyco-1–CTSD pathway analysis. For molecular and protein analysis, the left ventricular tissue was immediately frozen in liquid nitrogen and stored at −80°C until further processing. In addition, a portion of the tissue was fixed in 10% neutral buffered formalin for 24–48 h for histological and immunohistochemical analysis. Frozen tissue samples were subsequently homogenized for western blotting autophagy-related markers, while formalin-fixed samples were sectioned and stained to assess histopathological changes. This standardized sampling protocol ensured optimal preservation of left ventricular tissue for accurate biochemical and structural assessments.

The blood collected was transferred into microtubes for plasma separation, allowing for the analysis of lipid, insulin, glucose, and vitamin D3 levels. Blood samples were centrifuged at 3000 rpm for 15 min (Samsung 8-branch model, Korea) to separate the plasma, which was then stored in a freezer at −80°C for further research stages. The following formula was used to calculate HOMA-IR index [41]:

 $$\begin{array}{c} \text{HOMA}‐\text{IR}=\text{insulin }\left(\text{mmol}/\text{L}\right)*\text{glucose }\left(\text{mmol}/\text{L}\right)/22.5\;\left(\text{insulin assay}\right). \end{array}$$

To investigate the effects of HIIT and vitamin D3 on heart tissue in diabetic rats, the left ventricle was extracted and homogenized in liquid nitrogen. Proteins were separated on SDS-PAGE gels, transferred to PVDF membranes, and incubated with antibodies against Fyco-1 (N-18)-sc:79949, Beclin-1(E8)-sc:48341, mTOR (30):sc-517464, pmTOR (59.Ser 2448):sc-293133, and CTSD (C-5):sc-377124 (all, Santa Cruz Biotechnology, California, United States). After incubation with secondary antibodies, proteins were visualized using ECL Plus reagent. Immunoblots were then stripped and reprobed for mTOR, Beclin-1, Fyco-1, and total CTSD. Protein expression levels were normalized using *β*-actin as the loading control, ensuring consistent protein quantification across all samples.

Plasma glucose levels were measured using a colorimetric method, while insulin levels were determined via ELISA. Cardiac muscle tissue was homogenized in a buffer, centrifuged, and protein concentration was measured. The homogenate was prepared for western blot analysis by dissolving it in sample loading buffer and boiling to prevent nonspecific bands. This process enabled precise quantification and analysis of protein content related to autophagy and inflammatory markers in cardiac tissue.

### 2.7. Control of Possible Confounding Factors

In this study, confounding factors such as age, gender, baseline health status, circadian rhythm, fasting blood glucose levels, diet, and environmental conditions were controlled to ensure the accuracy and validity of the results. Only male Wistar rats between the ages of 6 and 8 weeks were included in the study, and they were randomly assigned to different groups using an online website. Interventions, such as HIIT and vitamin D3 injections, were performed at fixed times (early morning) to control for the effect of circadian rhythm. Fasting blood glucose levels after STZ injection ranged from 15 to 25 mmol/L, and values above 250 mg/dL were considered diabetic. All diabetic rats were fed a standard HFD (45% fat, 35% carbohydrate, 20% protein) that was constant throughout the study. Environmental conditions included a temperature of 20°C–24°C and a relative humidity of 40%–70% and were controlled with environmental monitoring systems to avoid sudden changes. These measures were taken to reduce stress and variability in physiological responses, as well as to reduce bias in the results. The blinding report in the present study was that the allocation of animals to different experimental groups was done by a person who was not involved in the study. The animal care stage and the execution of various experiments were done by researchers who were aware of the animal grouping, while the evaluation of the results and data analysis were done blindly so that these people had no knowledge of the grouping.

### 2.8. Statistical Analysis

Descriptive statistics, including mean, standard deviation, and percentage change in the mean, were used to describe the data in this study. The Shapiro–Wilk test was conducted to assess data normality. If the data showed normal distribution, a two-way ANOVA was performed, and Bonferroni's post hoc test was used to identify significant differences between groups when two-way ANOVA results were significant. To examine the hypothesis of homogeneity of variances, Bartlett's test was used. The results of this test showed that the variance of all groups was the same and homogeneity of variances was confirmed. All data analyses were conducted using GraphPad Prism software version 9 (United States) with a significance level of *p* < 0.05. Final analysis was performed for each experimental group (*N* = 8).

## 3. Result

Indicators of weight, glycemic control, and plasma vitamin D3 levels are presented in Table 2.

The results showed that there was a significant difference between the groups in mean weight (main effect HIIT: *F* (1, 36) = 205.88, *p* < 0.001; main effect VD3: *F* (1, 36) = 65.02, *p* < 0.001; and main effect interaction HIIT∗VD3: *F* (1, 36) = 4.51, *p* < 0.041). The results showed that in all three groups—DT, DV, and their combination—the mean weight was significantly lower than in the DC group (by 11.19%, *p* < 0.001; 4.37%, *p* < 0.03; and 22.62%, *p* < 0.001, respectively). The DTV group had significantly lower weight indices than the DT group by 12.87% and the DV group by 19.09% (*p* < 0.001). Additionally, the DT group had a 13.7% lower weight index compared to the DV group (*p* < 0.001). Both HIIT and vitamin D3 independently reduce weight significantly. The combined effect of HIIT and VD3 is significant but smaller than the main effects.

The results showed that there was a significant difference between the groups in mean glucose (main effect HIIT: *F* (1, 36) = 9.65, *p* < 0.004; main effect VD3: *F* (1, 36) = 9.13, *p* < 0.006; and main effect interaction HIIT∗VD3: *F* (1, 36) = 11.42, *p* < 0.001, *η*^2^ = 0.95). The results related to glycemic control indicators showed that plasma glucose levels were significantly higher in the DC group compared to the NC group (477.78%, *p* < 0.001). In all three treatment groups—DT, DV, and the combined DTV—the mean plasma glucose levels were significantly lower than in the DC group (29.38%, *p* < 0.001; 20.08%, *p* < 0.03; and 40.16%, *p* < 0.001, respectively). The DTV group also had significantly lower plasma glucose levels than the DT group by 15.26% (*p* < 0.01) and the DV group by 25.13% (*p* < 0.001). Additionally, the DT group had 11.64% lower plasma glucose levels compared to the DV group (*p* < 0.037). Both HIIT and vitamin D3 independently reduce glucose plasma significantly. The combined effect of HIIT and VD3 is significant but greater than the main effects.

The results showed that there was a significant difference between the groups in mean insulin serum (main effect HIIT: *F* (1, 36) = 3.34, *p* < 0.001; main effect VD3: *F* (1, 36) = 1.22, *p* < 0.009; and main effect interaction HIIT∗VD3: *F* (1, 36) = 7.31, *p* < 0.001, *η*^2^ = 0.87). Plasma insulin levels were significantly higher in the DC group compared to the NC group (104.45%, *p* < 0.001). In all three treatment groups—DT, DV, and DTV—the mean plasma insulin levels were significantly lower than in the DC group (by −29.75%, *p* < 0.001; −30.19%, *p* < 0.003; and −48.48%, *p* < 0.001, respectively). Insulin levels in the DTV group were also significantly lower than in the DV group by 47.27% (*p* < 0.001) and were 20.99% lower in the DT group compared to the DV group (*p* < 0.001). No significant difference was observed between the DT and DTV groups (*p* < 0.24). Both HIIT and vitamin D3 independently reduce insulin serum significantly. The combined effect of HIIT and VD3 is significant but greater than the main effects.

The results showed that there was a significant difference between the groups in mean HOMA-IR (main effect HIIT: *F* (1, 36) = 6.29, *p* < 0.001, *η*^2^ = 0.91; main effect VD3: *F* (1, 36) = 4.08, *p* < 0.009, *η*^2^ = 0.85; and main effect interaction HIIT∗VD3: *F* (1, 36) = 9.19, *p* < 0.001, *η*^2^ = 0.94). The results related to the HOMA-IR index showed that it was significantly higher in the DC group compared to the NC group (866.67%, *p* < 0.001). In all three treatment groups—DT, DV, and the combined DTV—the mean plasma glucose levels were significantly lower than in the DC group (−52.73%, *p* < 0.001; −46.12%, *p* < 0.03; and −66.67%, *p* < 0.001, respectively). These changes were significantly greater in the DTV group compared to the DT group, with a 28.89% reduction (*p* < 0.018) and a 50% reduction compared to the DV group (*p* < 0.001). In the DT group, plasma glucose levels were 29.69% lower compared to the DV group (*p* < 0.001). Both HIIT and vitamin D3 independently reduce HOMA-IR significantly. The combined effect of HIIT and VD3 is significant but greater than the main effects.

The results showed that there was a significant difference between the groups in mean VD3 serum (main effect HIIT: *F* (1, 36) = 111.7, *p* < 0.001, *η*^2^ = 0.94; main effect VD3: *F* (1, 36) = 121.18, *p* < 0.009, *η*^2^ = 0.96; and main effect interaction HIIT∗VD3: *F* (1, 36) = 133.07, *p* < 0.001, *η*^2^ = 0.97). The results related to the index of vitamin D3 plasma levels showed that it was significantly lower in the DC group compared to the NC group (31.10%, *p* < 0.001). In all three treatment groups—DT, DV, and the combined DTV—the mean plasma vitamin D3 levels were significantly higher than in the DC group (82.24%, *p* < 0.001; 127.49%, *p* < 0.001; and 52.15%, *p* < 0.001, respectively). These changes were significantly greater in the DTV group compared to the DT group, with a 40.21% increase (*p* < 0.001) and a 12.32% increase compared to the DV group (*p* < 0.014). In the DT group, vitamin D3 levels were 24.83% lower compared to the DV group (*p* < 0.001). Both HIIT and vitamin D3 independently increase VD3 serum significantly. The combined effect of HIIT and VD3 is significant but greater than the main effects.

The results of the ANOVA with repeated-measures analysis related to TTE showed that the effects of time (*p* < 0.001, *F* (4, 28) = 50.59, *η*^2^_time_ = 0.64), group (*p* < 0.026, *F* (4, 28) = 3.13, *η*^2^_group_ = 0.59), and the interaction of group by time (*p* < 0.001, *F* (4, 28) = 67.25, *η*^2^_group∗time_ = 0.73) were significant. TTE changes across different groups revealed that, in the DC group, there was a significant decrease in TTE at all measurement times (*p* < 0.001). In the D + HIIT and D + HIIT+VD3 groups, there was a significant increase in TTE at all measurement times (*p* < 0.001). This increase was significantly greater in the D + HIIT and D + HIIT+VD3 groups compared to the DC group (*p* < 0.001) (Table 3).

The changes in TTE were such that was significantly lower in the DC-post group compared to the NC-post group (*p* < 0.003, 13.13%). In the DT, DV, and interaction groups, the TTE was significantly higher than in the DC group (163.53%, *p* < 0.001; 93.46%, *p* < 0.001; and 197.49%, *p* < 0.001, respectively). These changes were significantly greater in the DTV group. Compared to the DV group, TTE was 53.77% higher (*p* < 0.001), and in the DT group, it was 36.22% higher than in the DV group (*p* < 0.001) (Table 3).

### 3.1. The Effect of HIIT, VitD3 Injection, and the Interaction of Both on the Protein Content of mTOR, pmTOR, and mTOR/pmTOR Ratio in the Left Ventricle

**Figure 2:**
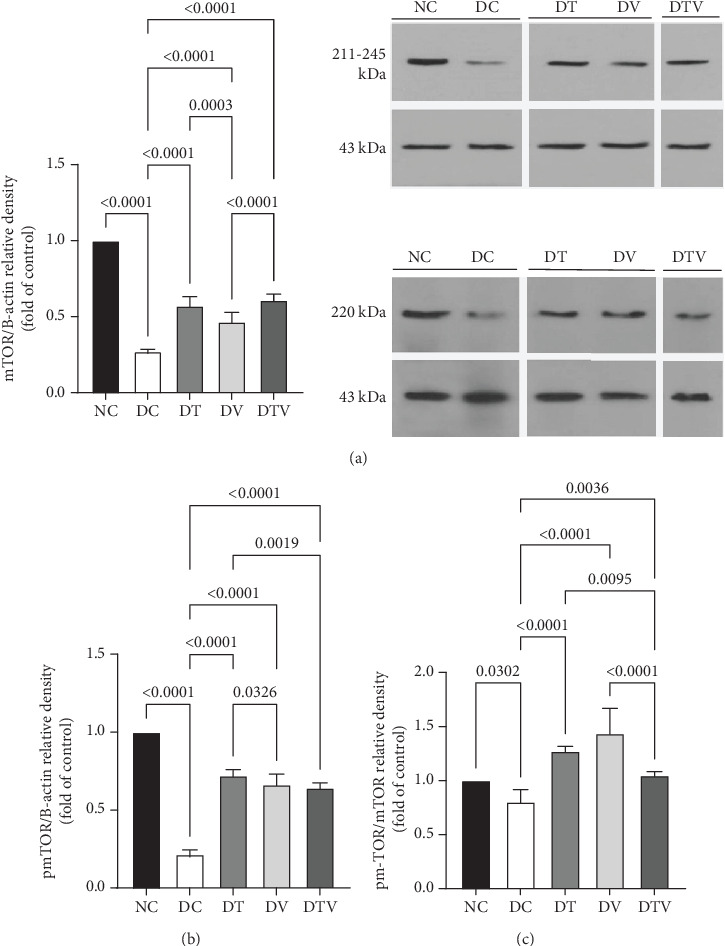
(a–c) The effect of HIIT, vitamin D3 (VitD3) injection, and their interaction on the protein content of mTOR, pmTOR, and the mTOR/pmTOR ratio in the left ventricle. Experimental groups: NC (normal control), DC (diabetes control), DT (diabetes + high-intensity interval training), DV (diabetes + VitD3), and DTV (diabetes + high-intensity interval training + VitD3). Data are presented as mean ± SD (*n* = 8). Statistical significance: *p* < 0.001.

The results showed that there was a significant difference between the groups in mean mTOR (main effect HIIT: *F* (1, 36) = 243.1, *p* < 0.001, *η*^2^ = 0.95; main effect VD3: *F* (1, 36) = 261.12, *p* < 0.009, *η*^2^ = 0.91; and main effect interaction HIIT∗VD3: *F* (1, 36) = 298.4, *p* < 0.001, *η*^2^ = 0.97), pmTOR (main effect HIIT: *F* (1, 36) = 434.5, *p* < 0.001, *η*^2^ = 0.96; main effect VD3: *F* (1, 36) = 451.33, *p* < 0.009, *η*^2^ = 0.89; and main effect interaction HIIT∗VD3: *F* (1, 36) = 487.15, *p* < 0.001, *η*^2^ = 0.97), and mTOR/pmTOR ratio (main effect HIIT: *F* (1, 36) = 36.33, *p* < 0.001, *η*^2^ = 0.95; main effect VD3: *F* (1, 36) = 43.13, *p* < 0.009, *η*^2^ = 0.92; and main effect interaction HIIT∗VD3: *F* (1, 36) = 30.07, *p* < 0.001, *η*^2^ = 0.97). The results showed that T2DM significantly decreased the protein content of mTOR (95% CI: 0.24–1, *p* < 0.03, 73%), pmTOR (95% CI: 0.19–1, *p* < 0.001, 79%), and the pmTOR/mTOR ratio (95% CI: 0.71–1, *p* < 0.03, 73%) in the DC group compared to the NC group. In contrast, the DT group exhibited a 111.1% increase (95% CI: 0.24–0.62, *p* < 0.001), the DV group 70.37% (95% CI: 0.24–0.51, *p* < 0.001), and the DTV group exhibited a 125.93% increase in mTOR protein content compared to the DC group (95% CI: 0.24–0.64, *p* < 0.001). Similarly, the DT group exhibited a 242.86% increase (95% CI: 0.19–0.75, *p* < 0.001), the DV group a 214.29% increase (95% CI: 0.19–0.71, *p* < 0.001), and the DTV group a 274.76% increase (95% CI: 0.24–0.66, *p* < 0.001) compared to the DC group. The pmTOR/mTOR ratio was also significantly increased in the DT group (95% CI: 0.71–1.07, *p* < 0.03, 58.75%), the DV group (95% CI: 0.71–1.63, *p* < 0.001, 78.75%), and the DTV group (95% CI: 0.71–1.07, *p* < 0.03, 31.25%) compared to the DC group. Additionally, there was no significant difference in the pmTOR/mTOR ratio between the DT and DV groups (*p* > 0.10), although the increase was higher in the DV group compared to the DT and DTV groups (*p* < 0.001) (Figures 2a, 2b, and 2c).

### 3.2. The Effect of HIIT, VitD3 Injection, and the Interaction of Both on the Protein Content of Beclin-1 in the Left Ventricle

**Figure 3:**
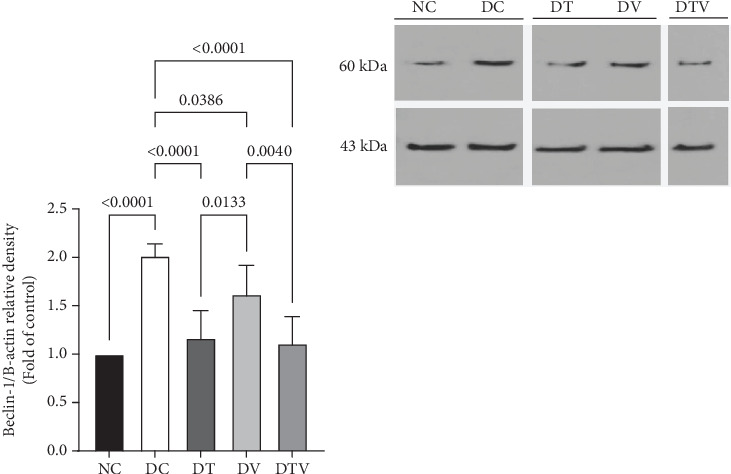
The effect of HIIT, vitamin D3 (VitD3) injection, and their interaction on the protein content of Beclin-1 in the left ventricle. Experimental groups: NC (normal control), DC (diabetes control), DT (diabetes + high-intensity interval training), DV (diabetes + VitD3), and DTV (diabetes + high-intensity interval training + VitD3). Data are presented as mean ± SD (*n* = 8). Statistical significance: *p* < 0.001.

The results showed that there was a significant difference between the groups in mean Beclin-1 (main effect HIIT: *F* (1, 36) = 24.7, *p* < 0.001, *η*^2^ = 0.62; main effect VD3: *F* (1, 36) = 18.09, *p* < 0.009, *η*^2^ = 0.53; and main effect interaction HIIT∗VD3: *F* (1, 36) = 29.41, *p* < 0.001, *η*^2^ = 0.69). ANOVA results showed that T2DM significantly increased Beclin-1 protein content in the left ventricle of rats with induced T2DM compared to the NC group (95% CI: 1–2.30, *p* < 0.001, 101%). In contrast, the protein content in the DT group (95% CI: 0.93–2.30, *p* < 0.001, 41.79%), DV group (95% CI: 1.37–2.30, *p* < 0.038, 19.40%), and DTV group (95% CI: 0.88–2.30, *p* < 0.001, 44.78%) was lower than that in the DC group. The greatest decrease was observed in the DT (95% CI: 0.93–1.87, *p* < 0.013, 27.78%) and DTV (95% CI: 0.88–1.87, *p* < 0.001, 31.48%) groups compared to the DV group. However, there was no significant difference between the DT and DTV groups (*p* > 0.99) (Figure 3).

### 3.3. The Effect of HIIT, VitD3 Injection, and the Interaction of Both on the Protein Content of Fyco-1 in the Left Ventricle

**Figure 4:**
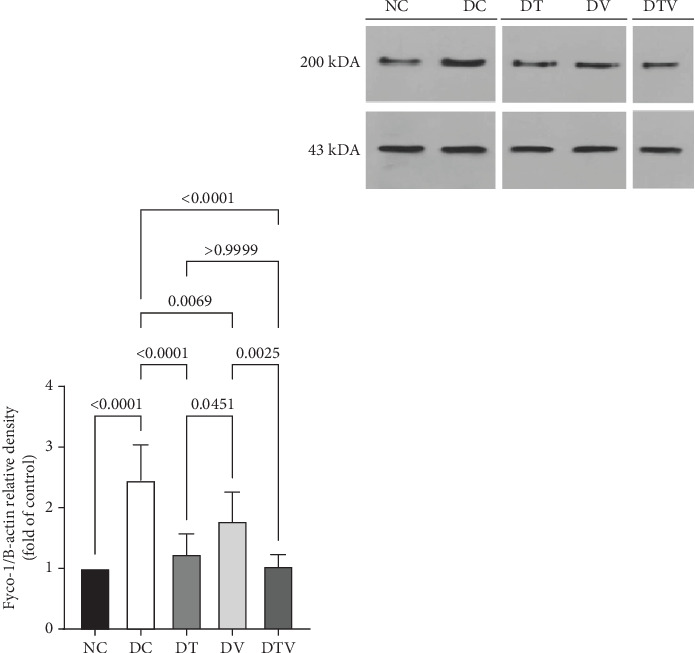
The effect of HIIT, vitamin D3 (VitD3) injection, and their interaction on the protein content of Fyco-1 in the left ventricle. Experimental groups: NC (normal control), DC (diabetes control), DT (diabetes + high-intensity interval training), DV (diabetes + VitD3), and DTV (diabetes + high-intensity interval training + VitD3). Data are presented as mean ± SD (*n* = 8). Statistical significance: *p* < 0.001.

The results showed that there was a significant difference between the groups in mean Fyco-1 (main effect HIIT: *F* (1, 36) = 29.16, *p* < 0.001, *η*^2^ = 0.72; main effect VD3: *F* (1, 36) = 21.59, *p* < 0.009, *η*^2^ = 0.63; and main effect interaction HIIT∗VD3: *F* (1, 36) = 34.62, *p* < 0.001, *η*^2^ = 0.78). Two-way ANOVA results showed that T2DM significantly increased Fyco-1 protein content in the left ventricle of rats with induced T2DM compared to the NC group (95% CI: 1–2.94, *p* < 0.001, 145%). In contrast, Fyco-1 protein content in the DT group (95% CI: 0.96–2.94, *p* < 0.001, 49.39%), DV group (95% CI: 1.38–2.94, *p* < 0.006, 27.35%), and DTV group (95% CI: 0.89–2.94, *p* < 0.001, 57.55%) was lower than in the DC group. The largest decrease was observed in the DTV group (95% CI: 0.89–2.18, *p* < 0.002, 41.57%) compared to the DV group, and the largest decrease in the DT group was observed compared to the DV group (95% CI: 0.96–2.18, *p* < 0.045, 30.34%). However, there was no significant difference between the DT and DTV groups (*p* > 0.99) (Figure 4).

### 3.4. The Effect of HIIT, VitD3 Injection, and the Interaction of Both on the Protein Content of CTSD in the Left Ventricle

**Figure 5:**
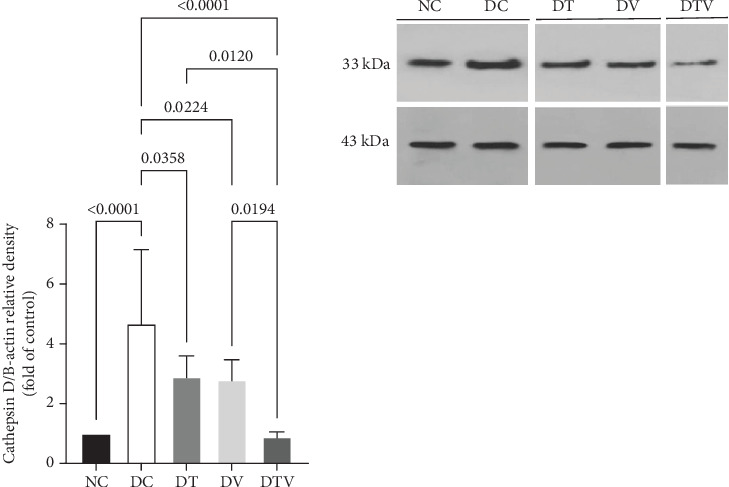
The effect of HIIT, vitamin D3 (VitD3) injection, and their interaction on the protein content of CTSD in the left ventricle. Experimental groups: NC (normal control), DC (diabetes control), DT (diabetes + high-intensity interval training), DV (diabetes + VitD3), and DTV (diabetes + high-intensity interval training + VitD3). Data are presented as mean ± SD (*n* = 8). Statistical significance: *p* < 0.001.

The results showed that there was a significant difference between the groups in mean CTSD (main effect HIIT: *F* (1, 36) = 14.26, *p* < 0.001, *η*^2^ = 0.61; main effect VD3: *F* (1, 36) = 16.45, *p* < 0.009, *η*^2^ = 0.63; and main effect interaction HIIT∗VD3: *F* (1, 36) = 18.23, *p* < 0.001, *η*^2^ = 0.69). The results showed that there was a significant difference between the groups in protein content CTSD (*F* = 13.99, *p* < 0.001, *η*^2^ = 0.61). The results of the ANOVA related to CTSD protein content showed that T2DM significantly increased the content of CTSD protein in the left ventricle of rats induced with T2DM compared to the NC group (95% CI: 1–2.94, *p* < 0.001, 59.18%). In contrast, in the DT group (95% CI: 2.33–6.74, *p* < 0.035, 37.82%), DV group (95% CI: 2.24–6.74, *p* < 0.022, 40.17%), and DTV group (95% CI: 0.76–6.74, *p* < 0.001, 80.98%), the protein content was lower than in the DC group. The largest decrease was observed in the DTV group compared to the DT group (95% CI: 0.78–3.46, *p* < 0.002, 69.42%) and also compared to the DV group (95% CI: 0.76–3.36, *p* < 0.002, 68.21%). There was no significant difference between the DT and DV groups (*p* > 0.99) (Figure 5).

## 4. Discussion

This study investigated the effect of vitamin D3 injection combined with HIIT on cell signaling pathways involved in excessive autophagy, specifically the mTOR–Beclin-1–Fyco-1–CTSD pathway, in the heart tissue of T2DM-induced rats. The findings revealed that T2DM significantly reduced the levels of mTOR, a key negative regulator of autophagy, and its pmTOR compared to the NC group. Notably, both HIIT and vitamin D3 injection effectively restored these protein levels, showing significant increases after 8 weeks of intervention. This is consistent with the results of our previous research, which demonstrated the increasing effect of MICT, with and without vitamin D3 supplementation, on the content of mTOR and pmTOR proteins [8]. However, the results of Lee et al. [42] were inconsistent, as they found that both HIIT and MICT training decreased mTOR protein levels in the hippocampal tissue of mice with T2DM. They also suggested that high-intensity exercise training, in combination with certain drugs, causes positive regulation of autophagy by reducing excessive activation of the PI3K/Akt/mTOR pathway. mTOR is a central regulator of cell growth and metabolism that inhibits autophagy under nutrient-rich conditions [43]. In T2DM, a decrease in insulin signaling disrupts mTOR activation, leading to lower levels of both mTOR and pmTOR [44]. This reduction likely removes the inhibitory effect on autophagy, thus promoting its regulation. Autophagy is a vital cellular process that degrades and recycles cellular components to maintain homeostasis. A key distinction between this study and Lee et al.'s research lies in the baseline autophagy activity of the animal models. While Lee et al. observed lower autophagic activity in diabetic hearts, our study reports elevated baseline levels of Beclin-1, a key initiator of autophagy, indicating excessive autophagic activity. Furthermore, increased levels of Fyco-1 and CTSD—proteins critical for autophagosome formation and maturation—support the presence of heightened autophagic activity in the diabetic heart [8, 9].

In T2DM, hyperglycemia and IR are key factors that affect cellular metabolism and signaling pathways. Elevated blood glucose levels lead to increased production of reactive oxygen species (ROS), which in turn causes oxidative stress [45]. The increase in ROS levels causes oxidative damage to cellular components such as lipids, proteins, and DNA, which likely stimulates autophagy in an attempt to remove and repair the damaged structures [46]. In addition, in chronic inflammatory conditions, the continuous activation of autophagy can lead to an imbalance between autophagy and cell death [47], ultimately resulting in excessive autophagy and cellular damage. However, to further confirm this, future studies can explore various aspects of the effects of excessive autophagy on oxidative stress and inflammation.

The results showed that the pmTOR protein content increased by 242.86%, 214.29%, and 204.6%, respectively, in the HIIT intervention, vitamin D3 injection, and the interaction of both, compared to the DC group. HIIT is known to activate multiple signaling pathways, including the mTOR pathway, through increased metabolic stress, mechanical load, and hypoxia. This leads to a strong stimulation of muscle protein synthesis and adaptation mechanisms specific to the intensity and nature of the training [48, 49]. In addition, vitamin D3 can indirectly affect the mTOR pathway by increasing calcium absorption and maintaining homeostasis. Alternatively, vitamin D3 may enhance mTOR signaling due to its anti-inflammatory properties [50]. It can be concluded that while pmTOR protein levels were significantly higher in all three intervention groups compared to the DC group, the increase was more pronounced in the vitamin D3 injection (DV) and HIIT (DT) groups than in the combined intervention (DTV) group. This finding suggests the possibility of a competitive effect or regulatory feedback mechanism when both interventions are combined, which may modulate mTOR pathway activation. As a result, the maximal stimulation of mTOR observed with either HIIT or vitamin D3 injection alone appears to be reduced in the combined intervention. Additionally, the levels of autophagy-related proteins (Beclin-1, Fyco-1, and CTSD) in the left ventricle of diabetic rats significantly decreased in all intervention groups compared to the DC group, with the largest reduction observed in the DTV group. These results highlight the interactive effect of the interventions in regulating autophagic activity and mTOR signaling. These findings indicate the positive effect of HIIT, vitamin D3, and their combined intervention in reducing excessive autophagy, which aligns with the results of previous research. Golpasandi et al. [8], Daryanoosh et al. [51] and Jokar et al. [52] reported consistent findings. Limited studies have investigated the effects of exercise training at different intensities on excessive autophagy levels. However, it can be said that exercise training can have a dual effect on impaired autophagy (either reducing or activating it). On the other hand, it can be stated that the activation of mTOR protein inhibits autophagy by blocking the formation of autophagosomes, thereby reducing excessive autophagy in heart tissue.

Various studies have reported the role of vitamin D3 in the regulation of autophagy [26, 53]. In the present study, the negative regulatory effects of vitamin D3 on autophagy levels were observed, which could be due to various possible mechanisms. As mentioned earlier, excessive autophagy can be associated with pathological conditions such as increased production of ROS (oxidative stress) or inflammation. In this regard, vitamin D3, likely due to its antioxidant and anti-inflammatory properties, may have reduced cellular signals related to inflammation and oxidative stress that contribute to excessive autophagy. This effect could be mediated through the activation of mTOR-related signaling pathways.

In the present study, the combined effect of HIIT and vitamin D3 injection effectively decreased the levels of Beclin-1, Fyco-1, and CTSD, indicating a significant reduction in autophagy. This combined approach not only targeted autophagy but also improved overall metabolic health, including better regulation of glucose levels, a reduction in the IR index, and an enhancement in physical performance. Specifically, the IR index was significantly lower in the DT and DV groups, as well as in the combined intervention group, compared to the DC group. This improvement is likely due to the effects of HIIT, vitamin D3 injection, or their interaction in increasing insulin sensitivity, improving glucose absorption, and ultimately reducing hyperglycemia [54, 55]. While this study provides significant insights into the regulation of autophagy by HIIT and vitamin D3 in a T2DM model, several research and practical limitations must be addressed before these findings can be confidently translated into clinical practice. Further research involving human subjects, a larger sample size, longer study duration, and consideration of individual variability and real-world conditions is necessary to confirm and optimize these interventions for the management of T2DM and its related complications.

The findings of this study show that T2DM significantly increases the levels of Beclin-1, Fyco-1, and CTSD proteins, which are important markers of autophagy activation and lysosomal activity. This increase suggests that autophagic processes are enhanced in diabetes as a maladaptive response to metabolic stress. At the same time, significant decreases in mTOR and pmTOR levels indicate disruption of anabolic signaling pathways that disrupt the balance of cellular homeostasis. Interventions including HIIT, vitamin D3 injection, and their combination were able to modulate hyperactive autophagy by reducing Beclin-1, Fyco-1, and CTSD levels and help restore balance. Also, the increase in mTOR and pmTOR levels indicates the reactivation of this important signaling pathway that plays a critical role in cell growth, protein synthesis, and metabolic regulation. These results overall highlight the biological importance of these interventions in correcting signaling abnormalities and improving metabolic and cellular health in T2DM.

## Figures and Tables

**Table 1:** HIIT protocol during 8 weeks.

**Week**	**High-intensity running**		**Low-intensity running**		**Work/rest ratio**	**Total exercise time in a session (min)**
	**Intervals/duration (min)/intensity % ** **V**_max_	**Training intensity according to running speed (m/min)**	**Intervals/duration (min)/intensity % ** **V**_max_	**Training intensity according to running speed (m/min)**		
First *V*_max_ test						
1	4%/2%/85%	20.83	4%/1%/50%	18.5	2:1	12
2	4%/2%/85%	20.83	4%/1%/50%	18.5	2:1	12
Second *V*_max_ test						
3	6%/2%/87%	20.92	6%/1%/50%	18.5	2:1	18
4	6%/2%/90%	21.05	6%/1%/50%	18.5	2:1	18
Third *V*_max_ test						
5	8%/2%/90%	21.05	8%/1%/50%	18.5	2:1	24
6	8%/2%/93%	21.18	8%/1%/50%	18.5	2:1	24
Fourth *V*_max_ test						
7	10%/2%/95%	21.28	10%/1%/50%	18.5	2:1	30
8	10%/2%/95%	21.28	10%/1%/50%	18.5	2:1	30
Fifth *V*_max_ test						

**Table 2:** Characteristics of weight, vitamin D3, and indicators related to glycemic control plasma levels.

	**Groups**				
**Variables**	**NC**	**DC**	**DT**	**DV**	**DTV**
Weight (g)	393.3 ± 11.93	375.3 ± 11.16	333.3 ± 10.69^$†‡^	358.9 ± 12.08^$‡^	290.4 ± 9.27^$†^
Glucose (mmol/L)	4.87 ± 1.43	22.36 ± 4.68^∗^	15.76 ± 1.06^$†‡^	17.87 ± 1.32^$‡^	13.38 ± 1.45^$†^
Insulin (mmol/L)	3.37 ± 0.05	6.97 ± 1.08^∗^	4.64 ± 0.10^$†^	4.81 ± 0.08^$‡^	3.55 ± 0.08^$†^
Vitamin D3 (ng/mL)	24.44 ± 2.08	16.84 ± 1.63^∗^	30.69 ± 4^$†‡^	38.31 ± 3.73^$‡^	43.03 ± 2.41^$†^
HOMA-IR	0.72 ± 0.19	6.96 ± 2.11^$^	3.29 ± 1.49^$†‡^	3.75 ± 1.14^$‡^	2.32 ± 0.79^$†^

*Note:p* < 0.001. D + HIIT: diabetes + high-intensity interval training, D + Vit D3: diabetes + vitamin D3 injection, D + HIIT + Vit D3: diabetes + high-intensity interval training + vitamin D3 injection.

Abbreviations: DC, diabetes control; NC, normal control.

⁣^∗^Significant versus the normal control group.

^$^Significant versus the diabetic control group.

^†^Significant versus the D + Vit D3 group.

^‡^Significant versus the D + HIIT + Vit D3 group.

**Table 3:** TTE changes at different times and in different groups.

		**Groups**				
**Variable**		**NC**	**DC**	**DT**	**DV**	**DTV**
TTE (second)	Pretest	20.15 ± 1.94	20.91 ± 1.89	20.78 ± 2.22	20.73 ± 2.80	20.85 ± 2.40
	Post 1	20.19 ± 1.24	19.02 ± 1.45^∗^	21.10 ± 2.16^$^	20.86 ± 2.15	21.59 ± 2.45^∗^^$†^
	Post 2	20.10 ± 1.49	17.53 ± 1.31^∗^	22.15 ± 2.57^∗^^$†^	21.05 ± 2.35^∗^^$^	23.57 ± 2.15^∗^^$†^
	Post 3	20.03 ± 1.10	16.62 ± 2.12^∗^	24.48 ± 2.08^∗^^$†^	21.18 ± 2.06^∗^^$^	26.23 ± 2.50^∗^^$†^
	Post 4	18.59 ± 1.75^∗^	11.16 ± 1.52^∗^	29.41 ± 2.31^∗^^$†^	21.59 ± 2.16^∗^^$^	33.20 ± 2.58^∗^^$†^

*Note: p* < 0.001.

Abbreviation: TTE, time to exhaustion.

⁣^∗^Significant compared to pretest.

^$^Significant compared to the DC group.

^†^Significant compared to the DV group.

## Data Availability

All data supporting the findings of this study are provided in the supporting information accompanying this article. The supporting information contains the original and full-length bands for each experimental group under different HIIT and vitamin D3 injection conditions. Furthermore, a detailed explanation of the blotting test procedures is included to ensure transparency and reproducibility.

## Supporting Information

Supporting Information Additional supporting information can be found online in the Supporting Information section. The supporting information includes complete western blot protein bands for each experimental group, along with a detailed explanation of the western blot measurement procedure. It provides additional data and methodological details that support the findings presented in the main text.
