# Supplementary material for: The Effect of Vitamin D3 Injection Combined With High-Intensity Interval Training on Excessive Autophagy in the Heart Tissue of Type 2 Diabetes–Induced Rats: An Analysis of the mTOR–Beclin-1–Fyco-1–Cathepsin D Pathway
Source: Cardiovasc Ther. 2025 Mar 19;2025:8817195. doi: 10.1155/cdr/8817195 (PMC11944841; doi:10.1155/cdr/8817195)
Supplement: Supporting Information — Additional supporting information can be found online in the Supporting Information section. The supporting information includes complete western blot protein bands for each experimental group, along with a detailed explanation of the western blot measurement procedure. It provides additional data and methodological details that support the findings presented in the main text. [file 8817195.f1.zip › Supplamentary file for Production.docx]

**Supplementary Materials: *Cardiovascular Therapeutics Journal*
Tittle:** The effect of Vitamin D3 Injection Combined with High-Intensity Interval Training on Excessive Autophagy in the Heart Tissue of Rats with Induced Type 2 Diabetes: An Analysis of the mTOR-Beclin-1- Fyco-1-CathepsinD Pathway.

**Table. 1, 2.** include the main and complete bands separately for each group in different interventions of high-intensity intermittent exercise training and vitamin D3 injection have been added, and the methods of how to implement the blotting test process have been presented in detail.

During the design phase of our research, we decided to investigate the effects of two types of exercise training: moderate-intensity continuous training (MICT) and high-intensity interval training (HIIT), combined with vitamin D3 injection. Consequently, in the western blot analysis, we categorized the samples based on the type of exercise regimen.

To accurately differentiate the effects of the two exercise protocols, we ensured that the bands for HIIT were clearly separated from those of MICT. In the present study, the placement of the blots has been meticulously separated to reflect this distinction. The highlighted sections in the table represent the specific parts utilized in the current research.

Additionally, we aimed to examine how vitamin D3 injections might interact with these distinct training protocols, potentially offering insights into their combined impact on physiological markers observed through the western blot method. This approach allows for a comprehensive understanding of how different exercise intensities, along with vitamin D3 supplementation, influence the biological responses under investigation.

(The highlighted parts in the table are the parts used in the present research), (Table 1,2).

**Table 1.** The shape of western blot bands of autophagy regulatory proteins and lysosomal network in the left ventricle of rats induced with type 2 diabetes.

|  | Normal  Control | Diabetic Control | Diabetes+ MICT | Diabetes+ HIIT | Diabetes+ VD3 | Diabetes+ VD3 + MICT | Diabetes+ VD3 + HIIT |
| --- | --- | --- | --- | --- | --- | --- | --- |
| mTOR | 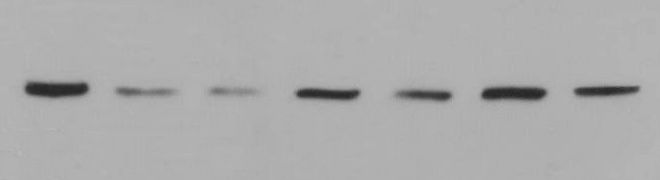 | | | | | | |
| B-actin | 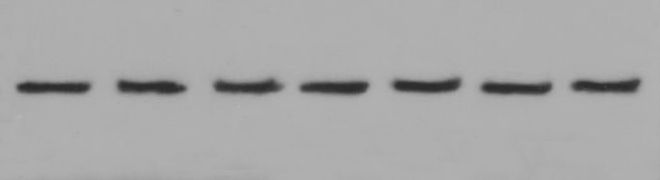 | | | | | | |
| pmTOR | 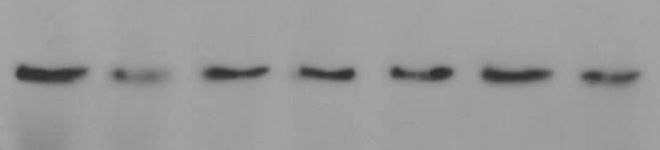 | | | | | | |
| B-actin | 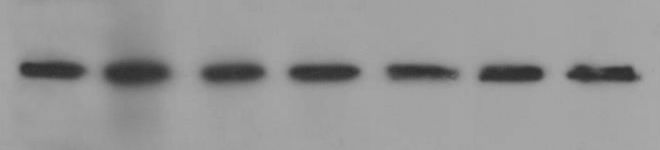 | | | | | | |
| Beclin-1 | 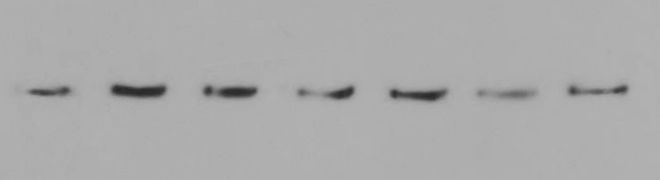 | | | | | | |
| B-actin | 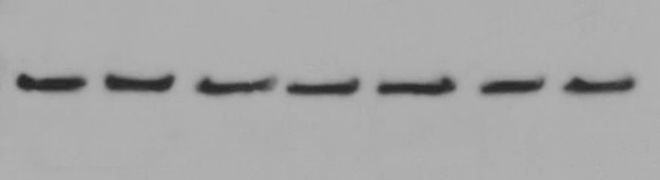 | | | | | | |
| Fyco-1 | 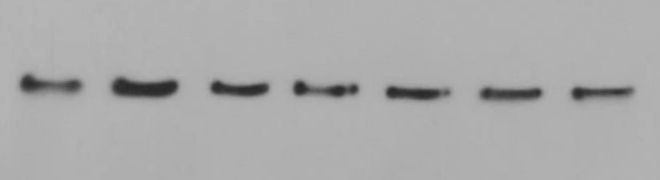 | | | | | | |
| B-actin | 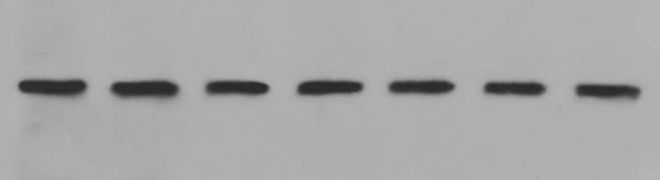 | | | | | | |
| Cathepsin D | 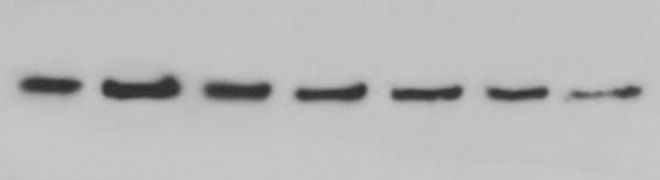 | | | | | | |
| B-actin | 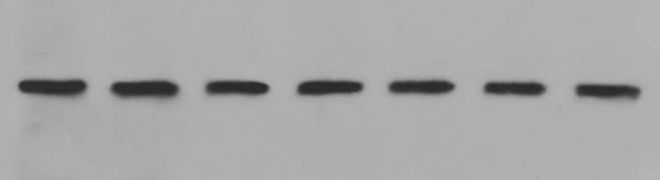 | | | | | | |

**Table 2.** The shape of the original western blot bands of autophagy and lysosomal network regulatory proteins in the left ventricle of rats induced with type 2 diabetes.

| 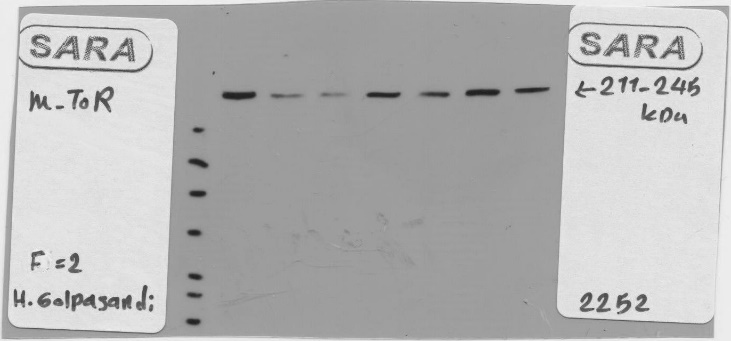 | 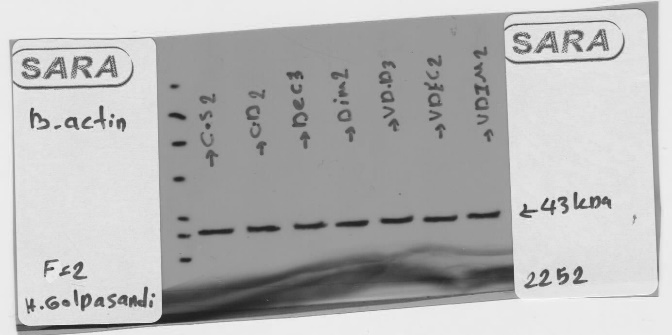 |
| --- | --- |
| 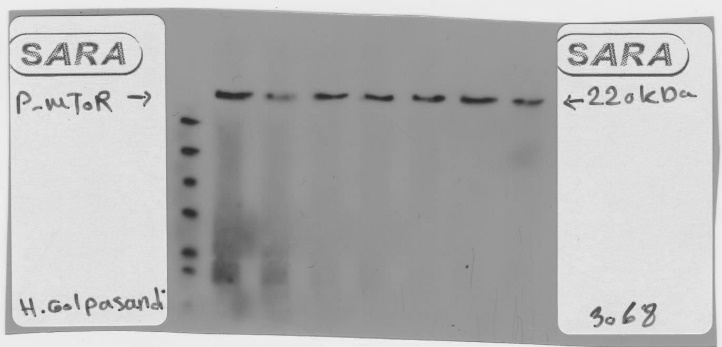 | 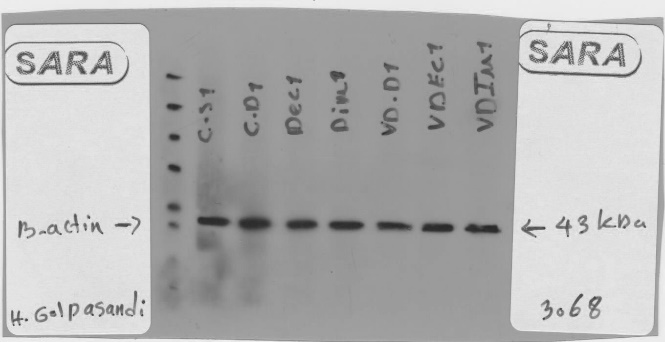 |
| 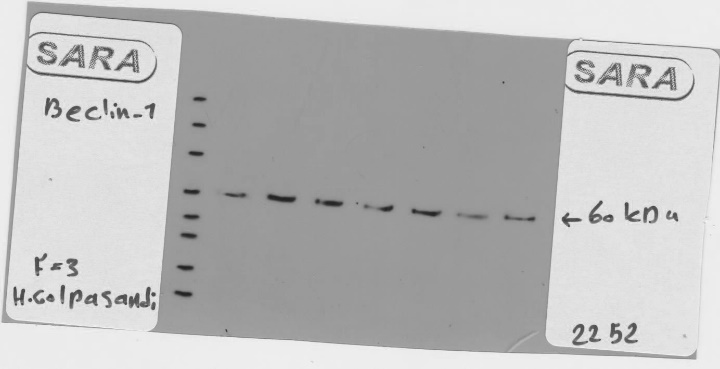 | 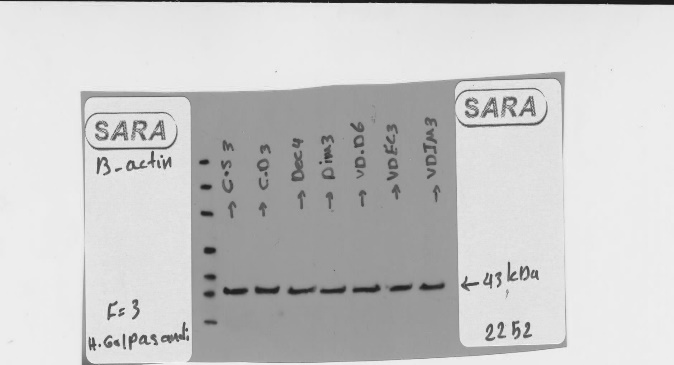 |
| 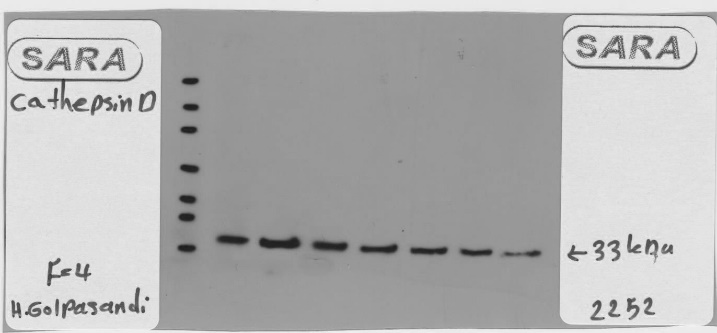 | 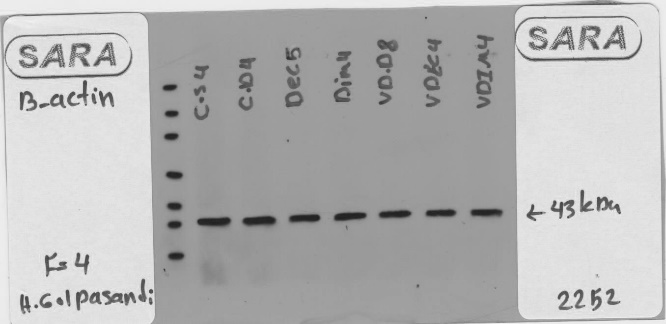 |
| 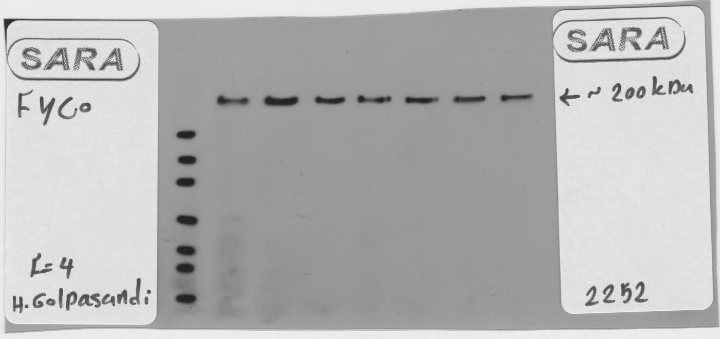 | 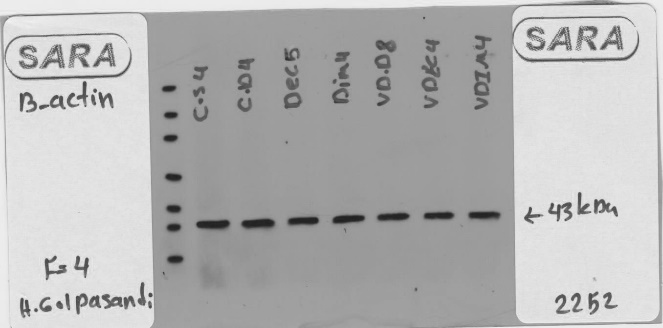 |

**Western blotting test**

- **Tissue lysis:**

Lysis buffer with the following composition was used to lyse the tissues (Table 3) and then the samples were centrifuged in Eppendorff 5415 R centrifuge at a temperature of four degrees Celsius and at a speed of 12000 rpm for 10 minutes. The clear liquid (supernatant) containing protein was extracted and stored in a freezer at -20.

**Table 3**. Tissue lysing buffer compounds.

| 500µL, PH=8 | Tris-HCL |
| --- | --- |
| 0.003gr | EDTA |
| 0.08gr | NaCl |
| 0.025gr | Sodium Deoxycholate |
| 0.01gr | SDS |
| 1tablet | Protease inhibitor cocktail |
| 10µl | NP40((1%)) Triton |

- **Determination of protein concentration by Bradford**

One of the methods for determining protein concentration is the Bradford method, which is based on the difference in light absorption at the wavelength of 630 in the device (Table 4). By increasing the concentration of the protein solution, the resulting blue color increases and its light absorption increases. The ingredients needed to make Bradford solution are listed in Table 2-3. To make Bradford Coomassie Blue solution, it is completely dissolved in alcohol for 20 minutes, then phosphoric acid is added drop by drop. Then we add water drop by drop until the volume of the resulting solution reaches 50 ml. The prepared solution is filtered twice with filter paper and stored in a dark bottle in the refrigerator.

**Table 4**. Ingredients needed to make Bradford solution.

| 5mgr | Comassie Blue G250 |
| --- | --- |
| 2.5mgr | Ethanol 95% |
| 5 mgr | Phosphoric acid |
| تا 50ml | Water Distilled |
| filter paper | Filter by |

- **Preparing different concentrations of BSA to draw a standard curve**

BSA is used as a standard protein to measure the amount of protein. Concentrations of 0.25, 0.5, 1, 0.15, 0.3, 0.6, 0.125 of the standard protein were created by adding half the volume of water to the previous concentration. In this way, 25 microliters of water were poured into all the wells of a row of flat bottom ELISA plates except the first one. Then, 50 microliters of BSA with a concentration of 1 mg/kg was poured into the first well, then 25 microliters were removed and poured into the second well. After complete mixing, we take 25 microliters from the second well and add it to the third well. This process continues until the end, but in the last well, 25 microliters is removed and discarded. The average optical absorbance of each concentration is used to draw a standard curve that shows the optical absorbance of protein with specific concentrations. The samples are poured into the wells in the amount of 25 microliters each with 3 repetitions. Finally, 200 microliters of Bradford's solution are poured into the wells, and the reaction of Bradford's solution with protein creates a blue color. Then the plate is placed in the ELISA reader and its OD optical absorbance is read at a wavelength of 630 nm for each well. In order to obtain the concentration of the desired protein, the standard curve is drawn from the optical absorption of BSA with certain concentrations using Excel software (Chart 1-3) and by obtaining the slope equation of the graph line so that R2 is between 1 and 0.99, the protein concentration The target is calculated according to its optical absorption in terms of mgr/ml.

- **Concentrations of protein, water and sample buffer**

It was determined by the average data of Bradford of each sample as well as the upper and lower data of the standard curve of protein concentration and subsequently water and sample buffer to make the sample. To increase the volume of the samples, they were all multiplied by 3.

- **Sample preparation**

Before being poured into the well, the prepared protein samples should be concentrated and mixed with the sample buffer and boiled in 100°C water for 5-10 minutes. This buffer causes proteins to become heavier, regenerated and linearized. In addition, the bromophenol blue present in the buffer shows the movement of proteins in the gel. The method of making the sample buffer is shown in **figure 1**.

**Figure 1.** Determination of concentration by Bradford for hippocampus.

- **Making electrofuge on SDS page gel**

SDS page gel is made of acrylamide polymer, which is cross-linked by bis-acrylamide. In such a way that pores with a certain and identical diameter are obtained in the gel. The polymerization of the gel started by adding ammonium persulfate (APS) and by adding tetramethyethylenediamine (TEMED) it caused the formation of free radicals from APS, and these radicals cause polymerization because free OH- in TEMED is needed to perform these steps, so at low pH Polymerization is completely stopped or slowed down. The polypeptides became negatively charged due to the presence of SDS, and the ratio of this charge to the molecular weight is the same for all the polypeptides in the sample, and in fact, the molecules are separated from each other due to the size of the gel pores and based on their size (Table 5-8).

**Table 5.** Creating a sample buffer (3X).

| 0.6ml | Tris (*PH=6.8) |
| --- | --- |
| 2.5mgr | Glycerol |
| 0.5mgr | ᵦ-mercapto ethanol |
| 0.01gr | Bromo phenol bule |
| 0.2gr | SDS |

- **Preparation of solutions**

**Table 6.** Acrylamide Stock solution (30.8%).

| 15gr | acrylamide |
| --- | --- |
| 0.4gr | Bais’s acrylamide |
| 50ml | water |

**Table 7.** Low gel buffer (PH=8.8).

| 9.1gr | Tris |
| --- | --- |
| 0.2gr | SDS |
| to 50 ml | water |

**Table 8.** High gel buffer (PH=-6.8).

| 3.5gr | Tris |
| --- | --- |
| 0.2gr | SDS |
| to 50 ml | water |

- **Western blot or immunoblotting**

Immunoblotting is a method in which the protein bands separated by gel electrophoresis (SDS-page) are transferred to a membrane made of nitrocellulose or PVDF, and then the proteins on it are identified by a specific antibody. Transfer of samples from gel to paper is done by electric current.

- **The transition stage from gel to paper**

After the electrophoresis, the gel is slowly separated from the glasses and placed in the transfer buffer. Then the PVDF paper is cut to the size of the gel and shaken in methanol for 1 minute to activate, washed with distilled water and placed in the transfer buffer. Transfer of protein from gel to paper is done in western blot machine. This device contains a tank containing a transfer buffer, which also contains a sponge, an ice container and a special cassette. Papers, sponge and gel are placed inside this cassette. The order of placement in the cassette includes sponge, filter paper, gel and PVDF paper, filter paper and sponge, and it is called a sandwich. While placing the filter paper on the PVDF paper and the PVDF paper on the gel, the bubbles created by the slow movement of the spacer on the filter paper are removed. Finally, the device is connected to the current generator with a voltage of 120 millivolts for one and a half hours and the proteins in the gel are transferred to the paper.

- **Blocking stage**

In the Blocking step, blocking solution is used to cover the paper to prevent non-specific reaction of the primary antibody. To make this solution, 2% of non-fat dry milk is added in TBS-T buffer, after the transfer of proteins on the PVDF paper surface, the paper is shaken with blotting solution for one hour and 15 minutes at room temperature. A very important point in this step is that the more the blocking solution of this step is used about 5-6 times, the less background of the blot is created in the films that appear.

- **Revealing stage**

Among the most accurate and sensitive techniques for detecting the desired protein band (identified by its specific primary antibody) is the use of Chemoluminescence kits. The ECL advanced reagents kit that was used in this study includes non-fat milk and Reagents A and B, and to detect Reagents A and B, they are mixed with a ratio of 1:1, and the final volume of the mentioned solution is 0.1 ml for each cubic centimeter of paper. is considered After the final washing of the previous step, the excess water of the PVDF paper is placed on the cellophane and the diminuluminescence solution is poured on the desired band areas with a sampler. The paper is wrapped in cellophane and inserted into the film cassette.

- **The appearance of the film in the dark house**

To observe the desired protein band, we put the photographic film on the nylon-coated paper and close the cassette. The duration of the film remaining in the cassette depends on the type of antibody and the intensity of the light seen from the protein band. In the case of bFGF and BDNF antibodies, 60 to 80 seconds and in the case of β-actin antibody, 10 seconds is the appropriate time. After removing the film from the cassette, we first place it in the pan containing the developing solution for 20 seconds until the bands appear. Then we wash the film in a pan of water for 20 seconds and then shake it in the proofing solution for 20 seconds. Then we wash the film again with running water and hang it with a clip to dry.

- **Striping method**

Using the blotting method, it is used again to detect the amount of another protein. The striping solution of table 9, primary, secondary antibody and ECL kit is washed from the blot and prepares the blot for the measurement of another protein. Place the blot in the striping solution for two to three minutes at 37°C to wash the antibodies and ECL from it. Then the blots are washed 2 times and each time for 5 minutes with TBS-T buffer and the work is repeated from the blocking stage with the desired protein antibody until the end. This method actually avoids repeating the test on a specific sample and is usually used to measure the level of β-actin. β-actin is one of the proteins whose expression level is constant in the cell. This protein is used to solve the error of loading proteins equally in the wells. If equal amounts of proteins are loaded in the gel wells, the bands obtained from β-actin will have the same density in all samples.

**Table 9.** Striping solution making.

| 208µl | Mercaptanol |
| --- | --- |
| 12.5ml | Tris,IM |
| 4gr | SDS |
| To 200ml | Water |
